# Emergency care utilization by refugee children compared to controls: A statewide database analysis

**DOI:** 10.1371/journal.pone.0318248

**Published:** 2025-02-06

**Authors:** Anna H. Abrams, Jan Leonard, Sarah E. Brewer, Janine Young, Kathleen M. Adelgais

**Affiliations:** 1 Section of Emergency Medicine, Department of Pediatrics, University of Colorado Anschutz Medical Campus, Children’s Hospital Colorado, Aurora, Colorado, United States of America; 2 Data Science and Biostatistics Unit, Department of Biomedical and Health Informatics, Children’s Hospital of Philadelphia, Philadelphia, Pennsylvania, United States of America; 3 Adult and Child Consortium for Health Outcomes Research and Delivery Science, University of Colorado, Children’s Hospital Colorado, Aurora, Colorado, United States of America; 4 Department of Family Medicine, University of Colorado Anschutz Medical Campus, Aurora, Colorado, United States of America; 5 Department of Pediatrics, University of California, San Diego; Rady Children’s Hospital, San Diego, California, United States of America; The World Bank Group, CANADA

## Abstract

**Background:**

Refugees face barriers to accessing healthcare despite provision of short-term services after arrival. Limited access to sustained primary care may lead to increased emergency department (ED) utilization and little is known regarding how refugee children access emergency care.

**Objective:**

To compare the proportion of ED claims and median level of service (LOS) between refugee children and general population controls in a statewide claims database.

**Methods:**

We conducted a retrospective cohort study of medical claims for patients aged 0 to 18 years old in a statewide claims database from 2014–2019. Refugee claims were identified using deterministic linkage of children with known refugee status. Procedure and diagnosis ICD9/10, Healthcare Common Procedure Coding System, and Current Procedural Terminology codes were obtained from the statewide database to indicate type of claim (ED vs outpatient) and LOS. Demographics were extracted from a data warehouse. Primary outcome was the number of ED claims per 1000 combined ED and outpatient claims. LOS was measured as a proxy for ED visit complexity. We compared demographics, frequency of claims, and median LOS using chi-square.

**Results:**

There were 5,590,808 total claims with 1,235,476 ED claims. Median number of ED claims per individual patient was the same between groups, however the proportion of claims related to an ED visit was significantly higher in the refugee population than the general population controls (244 vs 221, p = 0.001). Median LOS for ED claims was Level 3 (99283) and there was no difference between groups.

**Conclusion:**

Proportion of ED claims was higher in a refugee population compared to controls with no differences in LOS, indicating higher ED utilization among refugees for all acuity levels. Further study is needed to determine if healthcare disparities account for this difference and if population specific services may support the care of the refugee children.

## Introduction

More than 3 million refugees have been admitted to the United States (US) since the passage of the Refugee Act in 1980 [1]. Over one third of these refugees are estimated to be children less than 14 years old [1]. Recent conflicts such as in Afghanistan and Ukraine have resulted in cascading crises, leading to a record 37 million children displaced worldwide in 2022 [2]. Refugee children often arrive with unique health needs including unmanaged acute and chronic conditions [3]. Refugee children also have high burdens of mental health disorders, such as post-traumatic stress disorder, and are at high risk for physical, developmental, and behavioral health challenges [4].

In addition to increased health needs, refugee families often face numerous obstacles to accessing healthcare such as a lack of transportation, language barriers, and difficulty navigating the health system [5]. Navigating the healthcare system is identified as a high priority concern in one recent needs assessment of a refugee community [6]. Additionally, barriers to accessing care can lead to misdiagnoses, diagnostic delays, and decreased quality of care [7,8]. Barriers to care such as those experienced by this population may also lead to increased emergency department (ED) utilization for low acuity encounters [9]. Studies demonstrate poor health outcomes and increased ED utilization in other vulnerable subpopulations but there is a gap in knowledge of outcomes and unique needs within the refugee community [10,11].

Importantly, refugee resettlement initiatives designed to help refugees obtain access to housing, food, and other services also support the navigation of the United States healthcare system [12]. These programs include a limited period of health insurance, case management, English language classes, and other services. Access to these wraparound services makes refugees a unique population of patients and healthcare utilizers. Studies that examine refugee health typically emphasize overall healthcare utilization, outcomes, and best primary care screening practices [4,13–15], as opposed to other access points to healthcare such as the emergency department (ED). Of note, children comprise 40% of refugees worldwide [16] and represent a unique population with specific healthcare challenges. Most existing studies, however, focus on adult refugees [14,17,18] and therefore, little is known about healthcare utilization among refugee children.

Given the role of emergency departments as a safety net for those with difficulty navigating the healthcare system, it is important to understand overall ED utilization among refugees, and specifically emergency care utilization, for refugee children. Existing services target primary care establishments and ancillary services. Understanding the utilization patterns of refugee children, especially for acute care visits, could inform policies that will facilitate targeted resettlement initiatives that better support refugee families. This study aims to describe how ED utilization including frequency and acuity level of visits among refugee children compares to that of the general population. Our primary objective was to characterize refugee utilization of acute care services via claims analysis of the proportion of ED claims and median level of service (LOS) of refugee children compared with general population controls using a statewide claims database. We hypothesized refugee children would utilize the ED more often than general population controls and for lower LOS encounters given known barriers to healthcare.

## Methods

### Study design, setting, and population

This is a retrospective cohort study of all outpatient and emergency claims from patients 0 to 18 years old in a statewide medical claims database from 2014–2019. Refugee children were identified from a patient-centered medical home providing domestic medical exams (DMEs) and compared to general population controls. The DME usually occurs within 30–90 days of arrival to the United States and focuses on identifying acute health concerns, screening for conditions and infectious diseases specific to patient’s travel history, and familiarizing newcomers with the healthcare system [19]. The case population (refugee children) is defined as those who received a DME, confirming their refugee status. Refugee patients were eligible for inclusion if they had a documented DME, were identified in the claims database, and were <18 years old. Encounters not meeting eligibility criteria were excluded. This study was approved by the Colorado Multiple Institutional Review Board (#20-0301).

### Data collection

Refugee patients were identified in the hospital electronic health record (EHR) using both probabilistic and deterministic linkage. Linkage and extraction were performed by a local multi-institutional data warehouse that specializes in integrating and linking large scale clinical, regional, state, and national data sets thus creating a comprehensive longitudinal patient record. The data warehouse, managed by a research informatics team, includes EHR data from a university health system, children’s hospital, provider billing data, and -omics data from a center for personalized medicine. Specifically, patients were matched within the Colorado All Payer Claims Database (CAPCD) housed in the data warehouse, using unique person identifiers. Procedure and diagnosis ICD9/10 codes, Healthcare Common Procedure Coding System (HCPCS), and Current Procedural Terminology (CPT) codes were obtained from the CAPCD. Patient demographics were extracted from the multi-institutional data warehouse. All extracted data was stored in REDCap, a HIPAA compliant research capture database.

### Variables and outcomes of interest

The primary outcome of this study was the number of ED claims per 1000 combined ED and outpatient claims. Visits per 1000 is a standard metric of ED utilization [20]. This outcome was chosen given that the CAPCD is the state’s most comprehensive health care claims database and contains all claims from the majority of covered payers [21]. The secondary outcome was LOS of ED claims as a proxy measure of ED visit complexity or acuity. HCPCS and CPT codes were used to indicate type of claim (ED codes 99281-99285; outpatient codes 99201-99205). Encounters with the lowest level of complexity are classified as Level 1 (CPT 99281) with increasing complexity up to Level 5 (CPT 99285). We extracted demographic data including age in years, sex, and patient reported ethnicity (Hispanic, Non-Hispanic), Race, and primary language. Race, ethnicity, and primary language were obtained from discrete EHR data fields.

### Data analysis

Descriptive analysis included frequencies and proportions for categorical variables and median (IQR) for non-normally distributed continuous variables. Comparisons between the refugee and general population controls were made using chi square, Fisher’s exact, and Mann Whitney U tests. Data were analyzed in SAS (version 9.4, SAS Institute, Cary, NC).

## Results

During the study period, there were 5,590,808 total claims (3,334 refugee; 5,587,474 controls) including 1,235,476 ED claims (815 refugee; 1,234,661 controls). Table 1 describes the study population; the median age of patients was 7.2 years (IQR 2.5–13.6) and 52% were male, with no significant differences between the refugee population and the general population. Most prominently self-reported racial identity among refugees included Other/Multiracial (31%), Black (28%), and Asian (21%); closely mirroring the respective refugee population and general population within the state of Colorado [22]. Proportion of encounters with missing race and ethnicity demographic data was higher in the refugee cohort than the general population controls (Table 1). Overall, English was the primary language for 85.8% of general population controls compared to 29.1% of refugees. The most common non-English primary reported language among the refugee cohort was Arabic (36.4%) followed by Nepalese (14.0%). Other languages are shown in Table 1.

**Table 1:** Demographics.

	Total ED patients (n = 224,543)	Refugees (n = 151)	General population control (n = 224,392)	p-value
Sex				0.85
Male	117,271 (52.2%)	80 (53.0%)	117,191 (52.2%)	
Female	107,259 (47.8%)	71 (47.0%)	107,188 (47.8%)	
Ethnicity				<0.001
Hispanic	86,126 (38.4%)	1 (0.7%)	86,125 (38.4%)	
Not Hispanic	124,692 (55.5%)	133 (88.1%)	124,559 (55.5%)	
Missing	13,725 (6.1%)	17 (11.3%)	13,708 (6.1%)	
Race				<0.001
White	123,150 (54.8%)	11 (7.3%)	123,139 (54.9%)	
Black	22,323 (9.9%)	42 (27.8%)	22,281 (9.9%)	
Asian	5,103 (2.3%)	31 (20.5%)	5,072 (2.3%)	
Native Hawaiian/Other Pacific Islander	635 (0.3%)	1 (0.7%)	634 (0.3%)	
American Indian/Alaska Native	1,188 (0.5%)	0 (0%)	1,188 (0.5%)	
Other/More than 1 race	62,028 (27.6%)	48 (31.8%)	61,980 (27.6%)	
Unknown	10,116 (4.5%)	18 (11.9%)	10,098 (4.5%)	
Language				<0.001
English	192,624 (85.8%)	44 (29.1%)	192,580 (85.8%)	
Spanish	26,103 (11.6%)	0 (0%)	26,103 (11.6%)	
Other	5,743 (2.6%)	107 (70.9%)	5,636 (2.5%)	
Arabic	737 (12.8%)	39 (36.4%)	698 (12.4%)	
Nepali	692 (12.0%)	15 (14.0%)	677 (12.0%)	
Somali	678 (11.8%)	11 (10.3%)	667 (11.8%)	
Amharic	510 (8.9%)	2 (1.9%)	508 (9.0%)	
Burmese	501 (8.7%)	1 (0.9%)	500 (8.9%)	
Number of ED claims per person, median (IQR)	4 (2–7)	4 (2–6)	4 (2–7)	0.94

Table 2 summarizes all emergency department claims and level of service (LOS) during the study period by refugee status. Claims were measured as ED specific claims per combined ED and outpatient claims as described. The median number of overall claims (ED combined with outpatient claims) per patient during the study period was 4 for both groups (IQR 2–6 for refugees and 2–7 for the general population; Table 1). However, the number of ED claims/1000 in the refugee population was significantly higher than the general population (244 vs. 221, p = 0.001).

**Table 2:** Claims and level of service.

	Total ED claims (n = 1,235,476)	Refugee ED claims (n = 815)	Non-Refugee ED claims (n = 1,234,661)	p-value
Age at Encounter, median (IQR)	7.2 (2.5–13.6)	7.1 (3.8–11.2)	7.2 (2.4–13.6)	0.21
Emergency Department Level of Service				0.11
Level 1 (CPT 99281)	22,919 (1.9%)	5 (0.6%)	22,914 (1.9%)	
Level 2 (CPT 99282)	136,740 (11.1%)	91 (11.2%)	136,649 (11.1%)	
Level 3 (CPT 99283)	575,969 (46.6%)	388 (47.6%)	575,581 (46.6%)	
Level 4 (CPT 99284)	342,815 (27.8%)	233 (28.6%)	342,582 (27.7%)	
Level 5 (CPT 99285)	157,033 (12.7%)	98 (12.0%)	156,935 (12.7%)	
ED Claims/1000*	221	244	221	**0.001**

*ED claims per 1000 combined ED and outpatient claims.

Median level of service (LOS) for ED claims is demonstrated in Table 2. Levels of medical complexity range from the lowest classified as Level 1 CPT 99281) to the highest classified as Level 5 (CPT 99285). During the study period, median LOS was Level 3 (CPT 99283, average complexity) for refugees and non-refugees, comprising 47% of all encounters. All other LOS categories were similar between groups, including the highest complexity LOS (Level 5; 12% of refugee encounters, 12.7% of general population encounters).

## Discussion

Refugee children have unique medical needs as well as specific services designed for pediatric patients in contrast to their adult counterparts [23]. Our study sought to examine trends in healthcare utilization of refugee children with a focus on the need for emergency and acute care, an area which has not been previously described. This study found that overall claims per individual patient were similar between refugee children and general population controls, however the proportion of ED claims was higher in refugees. Our findings suggests that emergency departments may provide an important source of healthcare access for refugee children and families.

Prior studies of adult refugees reveal higher overall healthcare utilization than non-refugee immigrants and the general population [24,25]. One possible explanation for this is the mandated medical screening exam on arrival for all refugees which not only is included as a healthcare claim but connects a patient and family to healthcare services for future use. Many medical homes specializing in refugee health, including the one partnered with for this study, serve as primary care for refugee patients and families after initial DME screening in addition to providing any subspecialty referrals when needed and assistance with healthcare and insurance navigation. Prior research has demonstrated decreased ED utilization among patients with access to a patient centered medical home [26]. We would therefore expect a decreased proportion of ED visits among the refugee group in this study compared to controls. It will be important in future studies to determine if there are other services specific for the refugee population that may be beneficial to include in the medical home model for centers serving this population.

The lack of difference in overall claims in our study of refugee children may be multifactorial. Urgent care visits were not captured in this database which may underestimate overall utilization. The similar overall claims may also reflect refugee-specific resources that facilitate healthcare access. For example, reasons for low rates of utilization among non-refugee immigrants include lack of insurance and other barriers to care, including transportation [24]. Transitional refugee services such as a period of health insurance designed specifically for refugee patients may overcome some of these difficulties, thus equalizing healthcare utilization between the two groups.

Despite similar rates of overall claims per individual patient, there were significantly more ED claims per combined ED and outpatient claims in the refugee cohort compared to the general population. Prior studies that focus on either adult refugees or immigrants in general without specifying refugee status, show mixed results. Some reveal high acute care utilization early in the resettlement period [14,27], while others show lower rates of utilization compared to the general population [28,29]. There are numerous possible explanations for our findings of higher proportion of ED claims among refugee children. Refugee children have unique physical and mental health needs [30]. The guidance given at new arrival screenings to pediatric patients, limited availability to bring children to a clinic during office hours, or level or parental concern may all prompt more ED utilization by refugee families. There is significant evidence of higher disease burden including increased rates of both infectious diseases and mental health complaints in the refugee population [4,31], and the overall health status of immigrants and refugees may result in a higher need for acute care [14,27–29].

Disparities in access to care may also impact healthcare utilization patterns in the refugee community. A recent narrative review identified themes that influence ED utilization by pediatric immigrant and refugee patients including financial accessibility and health insurance, language barriers, health literacy, and transportation [32]. Many of these barriers impact other marginalized patient populations resulting in disparities in care and health outcomes (e.g., financial accessibility) but may be exacerbated in the refugee population due to lack of familiarity with the healthcare system. The ED is generally easy to find, eliminates the barrier of coordinating an appointment, is open outside of work hours, and does not require insurance coverage or up-front payment [33]. These factors make the ED often the most accessible medical care regardless of acuity for subpopulations facing numerous barriers such as refugees.

It is also possible the proportion of ED claims in this study is higher in the refugee population due to a lower number of outpatient claims. Some studies show similar rates of primary care utilization between refugees and controls [28], while others found refugee patients were more likely to utilize both primary care and ED care than non-refugee controls [34]. It will be important to determine if refugee children are presenting to the ED for concerns that could be managed by primary care providers in an outpatient setting, or if they require emergency care more often than controls. The similar LOS between groups in this study indicates the higher proportion of ED visits in the refugee population is not only due low acuity visits that may be more suited for primary care encounters. Future studies would benefit from patient or encounter level data as well as qualitive data to determine the reasons refugee children and families seek care in emergency departments.

LOS was examined as a proxy for visit complexity and therefore acuity. Studies show high rates of underlying chronic and acute medical conditions among refugees compared to a native born population [35,36]. While LOS is similar between groups, worse overall health status of this population may contribute to the higher rates of ED claims across all complexity levels. However, if overall health status is worse among refugee children, we would expect increased healthcare usage overall (with outpatient visits included) but combined claims were similar between groups. LOS may also be influenced by subjectivity of physician coding, variations in triage, and differences in healthcare-seeking behaviors between refugee and non-refugee patients. This variability in LOS due to factors unrelated to the encounter or patient acuity could make LOS a less accurate measure of visit complexity. A more granular analysis in the future including reasons for visits (chief complaints, discharge diagnoses) as well as alternate proxies for visit complexity [37] will be important to further interpret these findings.

The variability in studies of ED utilization of refugees indicates the importance of comparing subpopulations and regions of lower ED utilization to help identify effective outpatient services, transitional programs, or public health initiatives. Qualitative work to gain detailed insights on when, how, and why refugee patients access emergency services in addition to a more granular quantitative analysis would also help clarify these findings and guide future interventions.

## Limitations

This retrospective cohort study had several limitations. We defined refugee as patients receiving a DME at a single patient centered medical home. All outpatient and emergency claims from patients 0 to 18 years old in the CAPCD during the study period were included in the general population controls. Limited demographic data in CAPCD and lack of refugee status documentation limited our ability to confirm birth country, and immigration status for the control group. At present, most hospital systems and other large databases do not document these immigration-related data elements. Therefore, for the purposes of this study we must assume that those not receiving a DME at the refugee clinic may be included in the control group. It is possible this led to misclassification bias as we cannot confirm birthplace of those in the general population controls. If a significant number of controls are refugees, a comparison based on refugee status between the two groups cannot be made. However, given the extremely large number of overall claims and relatively low prevalence of refugees, it is unlikely that a small subset of refugees or other immigrants included in the general population controls would significantly alter results. In addition, there are specific robust services specifically for refugee resettlement that are lacking in the general immigrant community. If non-refugee immigrants are included in the control group, they likely have less access to some care than refugees. For future studies that seek to understand the refugee population or other subpopulations, it is important to gather more granular data to reflect the diversity within racial and ethnic populations. To mitigate potential misclassification bias in the future, it will be important to identify reliable proxies for refugee or immigration status in the control group.

In addition, refugees were identified through a single medical home. It is possible that differences in ED utilization are secondary to practice-specific resources, barriers, or interventions. However, the medical home partner in this study specializes in newcomer and immigrant heath, and includes a health services navigation program. In contrast to the results of this study, we would expect patients with access to these specialized services would access emergency care less often, Further studies should include refugee patients with varied outpatient care access and experiences. The use of a single center to identify refugee patients also limited the overall number of refugees in the study. We were unable to control for language barriers in this relatively small population, however this will be important in future studies to determine if tailored services aside from language concordant care (e.g., interpreters) are needed.

There were also limitations of the CAPCD. We were not able perform an encounter level analysis using available CAPCD claims data. The CAPCD is a statewide claims database and contains multiple claims associated with one encounter (e.g., physician fee, facility fee). We could not reliably determine a specific variable or set of variables that would accurately translate claims to encounter level data. Therefore, geographic breakdowns, country of origin groupings, chief complaints, and other variables were not available for this study. It will be important to analyze these data at the encounter level in the future to better understand utilization patterns.

In addition, urgent care visits are not captured in the CAPCD, which may lead to overall underreporting of care utilization by all groups. Inclusion of urgent care visits and other care settings (e.g., telehealth) in future studies would allow for a more comprehensive and nuanced analysis. A more granular analysis in the future including reasons for visits (chief complaints, discharge diagnoses), urgent care claims, and other variables will be important to further interpret these findings.

## Conclusion

Refugee children in the United States are a unique and important patient population with patterns of healthcare utilization must be better understood. We found the proportion of ED claims was higher in this cohort of refugee children compared to the general population controls with no differences in level of service in a statewide claims database. These results signify that not only are refugee children presenting to the ED for medical care at higher rates than general population controls, but these visits are not only for lower acuity complaints. Further studies are needed to confirm similar patterns at the encounter level and in larger refugee populations and determine if healthcare disparities (e.g., insurance status, access to language concordant care, etc.) account for this difference in utilization. Future analyses expanding on this work must include a qualitative exploration of the reasons refugee patients and families seek emergency care, barriers and facilitators to emergency and primary care, and identify opportunities to improve access for the refugee population to healthcare in non-emergency settings. The findings of this study and future qualitative analyses may facilitate community-informed policies that support the implementation of tailored services to support the care of the refugee children.
